# Human Early Placental Development: Potential Roles of the Endometrial Glands

**DOI:** 10.1016/j.placenta.2007.01.007

**Published:** 2007-04

**Authors:** G.J. Burton, E. Jauniaux, D.S. Charnock-Jones

**Affiliations:** a Department of Physiology, Development and Neuroscience, Physiological Laboratory, University of Cambridge, Downing Street, Cambridge CB2 3EG, UK; b Academic Department of Obstetrics and Gynaecology, Royal Free and University College, London, UK; c Department of Obstetrics and Gynaecology, University of Cambridge, Downing Street, Cambridge CB2 3EG, UK

**Keywords:** Placental development, Endometrial glands, Histiotrophe, Early pregnancy

## Abstract

There is strong evidence that the endometrial glands play a key role in regulating placental development in many domestic species, but their contribution in the human has largely been ignored once implantation is complete. Here we re-evaluate their role during the first trimester. Connections between the glands and the intervillous space have been observed from day 17 post-conception through to the end of the first trimester. In the absence of a maternal arterial supply to the early placenta it is believed that the carbohydrate- and lipid-rich secretions represent an important source of nutrients during the first trimester, and possibly the beginning of the second trimester. The secretions also contain a variety of growth factors that may regulate placental morphogenesis since their receptors are present on villous and extravillous trophoblast, and villous endothelial cells. Other components of the secretions may modulate immune responses and trophoblast invasion at the materno-fetal interface. We speculate that lactogenic hormones secreted by decidual cells and the syncytiotrophoblast may act in concert with human chorionic gonadotropin to stimulate the secretory activity of glandular epithelial cells during the first trimester. There is circumstantial evidence, but as yet no conclusive proof, that deficient glandular activity is associated with pregnancy failure in the human.

## 1 Introduction

The endometrium of all mammals is richly endowed with glands that open on to the luminal surface. Secretions from the glands play an essential role is sustaining the conceptus prior to implantation, and in some species they continue to contribute to materno-fetal transfer throughout pregnancy via specialised areas of the chorion termed areolae [1]. Their role in the human has largely been ignored once implantation is complete, for it has generally been assumed that the conceptus is removed from endometrial secretions during the invasive form of implantation. However, there is increasing evidence suggesting that the glands continue to function during the first trimester and early second trimester, that they deliver their secretions into the intervillous space, and that they may play important roles in modulating events at the materno-fetal interface. Hence, we re-evaluate the role of the glands in regulating human post-implantation placental development during early pregnancy, making comparisons with other species where appropriate.

The initial attachment of the human conceptus to the uterine epithelium takes place between the openings of adjacent uterine glands [2]. By the time implantation is complete at day 10–12 post-conception (pc) the chorionic sac is surrounded by a mantle of syncytiotrophoblast, in which spaces representing the forerunners of the intervillous space, the lacunae, are already present. As the mantle enlarges it erodes into branches of the capillary plexus that lies beneath, and parallel with, the uterine epithelium. As a result of this erosion, maternal erythrocytes are visible within the lacunae, although as Hertig and Rock commented they are surprisingly scarce [3]. They are also very palely stained compared to counterparts within maternal vessels, leading Hamilton and Boyd to suggest that circulation through the lacunae may be stagnant or that the erythrocytes are affected by secretions from the syncytiotrophoblast [2]. Any circulation can only be of a capillary nature at this stage of development, although it may be aided by uterine contractions and other forces [4].

**Fig. 1:**
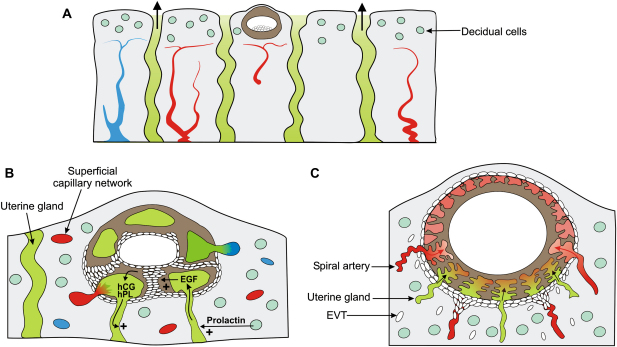
Diagrammatic representation of the relationship of the conceptus to the uterine glands during the first trimester. A) The blastocyst attaches and implants between openings of the uterine glands. The prevailing oxygen concentration will be low as the superficial decidua is oedematous and supplied by a capillary plexus arising from the spiral arteries, favouring trophoblast proliferation. B) As the conceptus enlarges the syncytiotrophoblast will invade into the superficial capillaries and the uterine glands, releasing the contents of both into the lacunae. EGF from the glands will stimulate trophoblast proliferation in the earliest stages, aiding development of the cytotrophoblastic shell. hCG and hPL from the syncytiotrophoblast, and prolactin from the decidual cells may in turn stimulate the glandular cells. C) As the conceptus enlarges the syncytiotrophoblast will encroach on the tips of the spiral arteries. It is essential that the cytotrophoblastic shell is well-formed by this stage, in order that sufficient endovascular extravillous trophoblast cells (EVT) are available to plug the spiral arteries beneath the conceptus. Incomplete plugging of the arteries in the periphery of the normal placenta is associated with early onset of the maternal circulation and villous regression to form the chorion laeve.

The expanding syncytiotrophoblastic mantle also erodes the epithelium of the adjacent uterine glands, and based on the density of the latter this is likely to be an early event. Thus, the openings of the glands on the non-pregnant uterine surface have an areal density of 15 per mm^2^ [5], and the chorionic sac of an embryo almost completely implanted into the endometrium, the ‘Barnes’ embryo estimated to be 11–12 day pc, measured 0.93 × 0.77 × 0.74 mm [2]. Destruction of the walls of the glands releases their secretions at the materno-fetal interface, and while some material disperses into the decidual extracellular matrix the bulk is delivered into the lacunae through channels formed in the cytotrophoblastic shell (Fig. 1). Connections between uterine glands and the developing intervillous space can be observed from approximately day 17 pc [6] throughout the first trimester [2,7].

The development of the endometrium that occurs during the secretory phase of the cycle appears to be maintained into early pregnancy. Measurements based on archival placenta-in-situ hysterectomy specimens indicate that the endometrium beneath the conceptus is 5–6 mm thick at 6 weeks gestational age (4 weeks pc), but reduces to 1–2 mm by the end of the first trimester [8]. Histologically, the glandular epithelial cells appear highly active during early pregnancy, and resemble those of the early secretory phase of the cycle [7–9]. They have a tall columnar phenotype, with accumulations of glycogen in the apical cytoplasm and of lipid droplets towards their base. By the end of the first trimester these cells are more cuboidal in shape, and appear morphologically to be more quiescent. These changes correlate with concentrations of glycodelin A, one of the principal components of the glandular secretions, which peak in the maternal serum and amniotic fluid towards the end of the first trimester and then fall rapidly [10,11]. Thus, it seems that in the human the contribution from the decidual glands is phased out in concert with the onset of the maternal intraplacental circulation [12]. Whether these two processes are co-ordinated, and if so how, is not known at present.

## 2 The endometrial-decidual glands as a source of nutrients

The composition of the secretions from the endometrial glands during the various phases of the menstrual cycle has been studied extensively [10,13], but the full range of secretory products during early pregnancy is not known. The secretions will be derived from two principal sources; a serum transudate arising from the rich capillary plexus surrounding the glands, and specific proteins, carbohydrates and other metabolites synthesised within the glandular cells. Quantitatively, the major glandular product detected in maternal serum is a dimeric glycoprotein that is now referred to as glycodelin A, but has been termed PP14 and α_2_-PEG in the past. Histochemistry confirms that secretions within the lumens of the glands are carbohydrate rich, for they react strongly with periodic acid Schiff reagent. They also contain numerous lipid droplets that stain bright red with Neutral Red dye [8]. Evidence that the glands may act as a source of nutrition for the conceptus is provided by the observation that the syncytiotrophoblast covering the surfaces of villi facing the endometrium contain large accumulations of glycogen that appear as crimson arcs on PAS-staining [7,14]. These accumulations are greatest close to the materno-fetal interface, suggesting a concentration-dependent uptake by the trophoblast.

In addition, during the first trimester the syncytiotrophoblast has been shown to phagocytose maternal glycoproteins, including glycodelin A [7]. Since the mRNA encoding glycodelin A is not expressed in placental tissues [15], the presence of vesicles immunoreactive for the glycoprotein within the syncytiotrophoblast confirms uptake. The staining is punctate, and in the mid-zone of the syncytioplasm it co-localises with cathepsin-D indicating that they are entering the lysosomal digestive pathway [8]. We speculate that maternal proteins may be phagocytosed non-selectively by the trophoblast, broken down and their constituent amino acids recycled in anabolic pathways in a fashion analogous to the yolk sac of the mouse. This pathway is reported to account for approximately 90% of amino acid uptake by the murine conceptus during the period of organogenesis [16], but as yet there is no conclusive evidence that this is the case in the human trophoblast.

The glands may also act as an important pathway for the transfer of micronutrients during early pregnancy, for we have observed expression of the α-tocopherol transfer protein in the glandular epithelial cells by immunohistochemistry during the first trimester [17]. This protein facilitates passage of tocopherols through epithelia, and had previously only been observed in the liver. Its presence in the glandular cells, on the surface of the syncytiotrophoblast and the outer surface of the secondary yolk sac suggests that the decidua may be an important source of antioxidants during the critical phase of organogenesis.

## 3 The endometrial-decidual glands as a source of growth factors

Besides providing a source of nutrients the decidual glands may play a more active role in regulating placental morphogenesis though the production of growth factors. A variety of growth factors have been identified within the glandular epithelium and the luminal secretions by immunohistochemistry, including epidermal growth factor (EGF), vascular endothelial growth factor (VEGF) and leukaemia inhibitory factor (LIF) [8]. Receptors for all these are present on the placental tissues during the first trimester.

The distribution of EGF-R varies with gestational age, being expressed only by cytotrophoblast cells at 4–5 weeks, and then by the syncytiotrophoblast alone at 6–10 weeks. There then follows a rapid decline in immunoreactivity, which remains low to term [18]. Addition of exogenous EGF to villous explants shows an equivalent biphasic action with gestational age. Thus at 4–5 weeks EGF stimulates cytotrophoblast cell proliferation, whereas at 6–12 weeks it stimulates secretion of human chorionic gonadotropin (hCG) and human placental lactogen (hPL) into the supernatant [19]. A similar action of EGF on trophoblast proliferation has been reported in the horse, where in situ hybridisation has demonstrated that the concentration of mRNA encoding EGF increases in the glandular epithelial cells during early pregnancy, except in areas of intense lymphocytic infiltration into the decidua. Proliferation is observed in the trophoblast overlying the mouths of the glands expressing EGF, but not in those negative for the mRNA [20]. A high rate of proliferation of cytotrophoblast cells is observed during human early pregnancy, and is necessary not only to generate the syncytiotrophoblastic covering of the expanding villous tree, but also to feed the cytotrophoblastic cell columns (Fig. 1). These merge at their distal ends to form the cytotrophoblastic shell that surrounds the conceptus and represents the materno-fetal interface during early pregnancy [2]. It is well-developed by day 17 pc [6], but begins to thin from day 36 pc onwards [2].

It is essential that the shell be fully developed before the expanding conceptus reaches the tips of the spiral arteries in the mid-zone of the functionalism, in order to protect the conceptus from the full force of arterial inflow at too early a stage of pregnancy [21]. The endovascular extravillous trophoblast cells are derived from the outer surface of the shell, and these cells migrate down the lumens of the spiral arteries, initiating their physiological conversion in the process. In the earliest stages the volume of endovascular trophoblast cells migrating into the arteries is such that their lumens are effectively blocked or plugged [21–24]. Failure of the shell to develop fully is associated with early onset of the maternal circulation to the placenta and failure of the pregnancy [25,26], probably as a result of incomplete plugging of the arteries [12]. This will lead to haemodynamic disturbances at the materno-fetal interface, and to excessive oxidative stress of the placental tissues. Each spiral artery supplies an area of uterine luminal surface of 4–9 mm^2^ [27], and so the chorionic sac must enlarge considerably before many will be encountered.

Receptors for VEGF and LIF have been identified on the villous and extravillous trophoblast populations, and also on villous endothelial cells [28–30]. These factors may play important roles in regulating placental angiogenesis, for mice lacking the LIF-R gene display altered vascular development.

## 4 Immunomodulatory and other actions of endometrial-decidual proteins

The decidual glands are known to produce a number of glycosylated glyoproteins, which may exert a diverse variety of effects as they diffuse into the extracellular matrix at the materno-fetal interface following erosion of the glandular epithelium. Glycodelin A has been attributed with an immunomodulatory role for it is able to suppress cytotoxicity of uterine Natural Killer (NK) cells in a dose-dependent fashion in vitro [31]. It has also been reported to reduce the secretion of interleukin-1 by activated lymphocytes in a similar dose-dependent fashion [32], and is a direct inhibitor of T-cell proliferation [33]. Hence, glycodelin A may play a role in regulating interactions between the NK cells and the invading extravillous trophoblast cells within the placental bed, in addition to the possible nutritional role described earlier.

An immunomodulatory role has also been suggested for uteroglobin, an unusually small globular protein that is produced in maximal quantities during the secretory phase and is a potent inhibitor of neutrophil and monocyte chemotaxis in vitro [34,35]. Uteroglobin also has other potentially significant actions, however, as it is able to reduce the invasiveness of trophoblast cell lines through Matrigel by binding to a novel receptor site [36]. Hence, it may have a more direct effect on regulating extravillous trophoblast invasion into the decidua.

Other proteins produced by the glands may play a role in the innate immune defences against infection. Thus, two families of natural antimicrobials, the defensins and the whey acidic protein (WAP) motif proteins, are found in the uterine and glandular epithelia [37]. Although each defensin has a unique temporal expression during the cycle, that of the WAP protein SLPI occurs during the mid-late secretory phase. Lactoferrin, which is weakly expressed in the glandular epithelial cells at 6 weeks gestational age [8], has been shown to act in synergy with SLPI as an antimicrobial in the lung [38]. Together, these components of the secretions may therefore play an important role in preventing infection during implantation and early pregnancy.

## 5 Regulation of glandular activity during early pregnancy

In the normal non-pregnant cycle the glycogen accumulations within the glandular epithelial cells begin to disperse around days 23–24, suggesting a decline in secretory activity [39]. The persistence of these accumulations through to at least 6 weeks indicates that the secretory stimulus is maintained into early pregnancy. Indeed, evidence from domestic species, including the sheep, rabbit and pig, indicates that the conceptus is able to signal to the glands and enhance their development and activity [40]. Thus, in the sheep the glands undergo considerable hyperplasia between days 15 and 50 pc, followed by hypertrophy to increase their surface area [41]. Whether there is a similar pattern of glandular development in early pregnancy in the human is uncertain, although Demir and colleagues did detect immunoreactivity for proliferating cell nuclear antigen (PCNA) in the glandular epithelium in their early pregnancy samples [9].

In the sheep, sequential exposure to oestrogen, progesterone, interferon tau, placental lactogen and placental growth hormone stimulate expression of uterine milk proteins in the glandular epithelial cells [42]. Interferon tau activates the JAK-STAT pathway within the cells, while binding of placental lactogen promotes formation of STAT5 homodimers. Both pathways promote expression of the uterine milk proteins, but downregulation of the progesterone receptor is also required as this exerts an inhibitory influence [40]. It is notable that the concentration of mRNA encoding the prolactin receptor increases during early pregnancy in the glandular epithelial cells alone, most likely in a paracrine response to placental lactogen coming from the trophoblast binucleate cells [43]. Prolactin also stimulates progesterone-induced uteroglobin expression in the rabbit uterus [44].

**Fig. 2:**
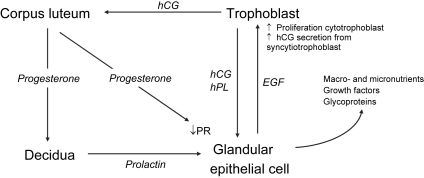
Summary of the potential servomechanism by which the human conceptus may stimulate activity in the glandular epithelial cells to meet its requirements.

It is possible to envisage an equivalent pathway operating in the human (Fig. 2). Thus, progesterone receptors cannot be detected on the glandular epithelial cells by immunohistochemistry during early pregnancy, consistent with their downregulation by exposure to progesterone during the late secretory phase of the cycle [45]. Progesterone receptor A (PR_A_) continues to be expressed by the decidual cells, however. Equally, the glandular epithelial cells contain the highest concentration of luteinising hormone/hCG receptors of all cells in the human uterus [46]. hCG plays an equivalent role to interferon tau in the sheep during early pregnancy, and treatment of isolated glandular cells with highly purified hCG results in a time- and dose-dependent increase in levels of cyclooxygenase-2 mRNA, protein and secretion [46]. The prolactin receptor is strongly expressed by glandular epithelial cells and decidualised endometrial stromal cells during the mid to late secretory phase, and during early pregnancy [47].

Thus in early human pregnancy hCG secreted by the syncytiotrophoblast acts through LH receptors to maintain the corpus luteum of pregnancy, ensuring that progesterone concentrations remain high. The continued exposure to progesterone will keep progesterone receptors on the glandular cells downregulated, thereby removing their inhibitory influence on the expression of milk proteins. The progesterone will also stimulate the decidual cells via the PR_A_ to secrete prolactin [48]. Within the placenta the syncytiotrophoblast also secretes human placental lactogen (hPL), which shares 67% sequence homology with prolactin and is able to signal through the prolactin receptor. These two lactogenic hormones, prolactin and hPL, may stimulate the glandular epithelial cells in concert with hCG also coming from the placenta. In this way the conceptus would be able to regulate the supply of nutrients and other factors it receives from the mother, which is an attractive hypothesis teleologically. Some evidence that this may be the case is provided by the fact that in baboons exogenous or blastocyst-secreted chorionic gonadotropin is able to stimulate the secretion of glycodelin [49,50].

In addition to these endocrine loops it is possible that local paracrine signals within the decidua influence glandular activity. Thus, it is known that uterine NK cells accumulate in the vicinity of the glands during early pregnancy, and some come to lie in close apposition with the epithelial basement membrane [8]. NK cells secrete a variety of cytokines when activated [51], and we have demonstrated that they are also immunoreactive for EGF. This raises the possibility that extravillous trophoblast/NK cell interactions may also signal the presence of a conceptus to the endometrial glands.

## 6 The impact of deficient glandular activity in early pregnancy

There has been much speculation in the past that deficient glandular activity, usually described as luteal phase defect, results in early pregnancy failure. Although there is considerable circumstantial evidence that this may be the case, there is as yet no conclusive evidence to support this claim in the human. Ultrasonographic studies have indicated that an endometrial thickness of at least 8 mm is necessary for successful implantation [52], although not all studies have supported this assertion [53]. Biochemical assessments of glandular activity have shown that concentrations of glycodelin A in uterine flushings on days LH + 10 and LH + 12 are lower in women who go on to miscarry than those with successful pregnancies [54]. Similarly, the concentrations of MUC-1, a progesterone-dependent glycoprotein secreted by the glands, are lower at day LH + 10 in women suffering recurrent spontaneous miscarriage than in fertile controls [55]. These findings are consistent with the observed failure of downregulation of progesterone receptors in glandular epithelial cells in women with luteal phase defect [56]. Such failure may mean that the normal inhibition of expression of uterine milk proteins exerted by the progesterone receptor is not removed, compromising the secretory activity of the glands in early pregnancy. Whether miscarriage in these cases is the result of an incomplete cytotrophoblastic shell secondary to inadequate EGF-induced proliferation, or due to abnormal immunological interactions within the decidua through a lack of modulation by glycodelin A, is not known at present.

More direct evidence of the importance of the endometrial glands for survival and normal development of the conceptus is provided by experimental models in the sheep where the glands can be ablated by administering 19-norprogestin for a period of 8 weeks immediately after birth. Uteri display a spectrum of responses to this treatment, with some having no glands and others appearing relatively normal. Conceptus survival and elongation was directly related to the degree of glandular development [57]. In the worst cases no conceptus was present on day 14, whereas in intermediate uteri a single non-expanded conceptus was present. Although it is potentially dangerous to extrapolate between species, we speculate that cases of biochemical pregnancies that fail to progress to clinical pregnancies and cases of empty gestational sacs represent equivalent outcomes of glandular dysfunction in the human. Further research is required to confirm this hypothesis, but the concept opens potential avenues for therapeutic interventions.
